# Aurora-A inactivation causes mitotic spindle pole fragmentation by unbalancing microtubule-generated forces

**DOI:** 10.1186/1476-4598-10-131

**Published:** 2011-10-19

**Authors:** Italia A Asteriti, Maria Giubettini, Patrizia Lavia, Giulia Guarguaglini

**Affiliations:** 1 Institute of Molecular Biology and Pathology, CNR National Research Council, c/o Department of Biology and Biotechnologies, Sapienza University of Rome, Via degli Apuli 4, 00185, Rome, Italy

**Keywords:** Aurora-A, mitotic spindle forces, multipolar spindles, ch-TOG, Eg5, Nuf2, MLN8237

## Abstract

**Background:**

Aurora-A is an oncogenic kinase playing well-documented roles in mitotic spindle organisation. We previously found that Aurora-A inactivation yields the formation of spindles with fragmented poles that can drive chromosome mis-segregation. Here we have addressed the mechanism through which Aurora-A activity regulates the structure and cohesion of spindle poles.

**Results:**

We inactivated Aurora-A in human U2OS osteosarcoma cells either by RNA-interference-mediated silencing or treating cultures with the specific inhibitor MLN8237. We show that mitotic spindle pole fragmentation induced by Aurora-A inactivation is associated with microtubule hyperstabilisation. Silencing of the microtubule-stabilising factor ch-TOG prevents spindle pole fragmentation caused by inactivation of Aurora-A alone and concomitantly reduces the hyperstabilisation of microtubules. Furthermore, decreasing pole-directed spindle forces by inhibition of the Eg5 kinesin, or by destabilisation of microtubule-kinetochore attachments, also prevents pole fragmentation in Aurora-A-inactivated mitoses.

**Conclusions:**

Our findings indicate that microtubule-generated forces are imbalanced in Aurora-A-defective cells and exert abnormal pressure at the level of spindle poles, ultimately causing their fragmentation. This study therefore highlights a novel role of the Aurora-A kinase in regulating the balance between microtubule forces during bipolar spindle assembly.

## Background

The assembly of a bipolar mitotic spindle is a highly dynamic process essential for balanced chromosome segregation in mitosis. Defects in the spindle bipolar architecture can cause unequal chromosome segregation to daughter cells and represent a source of aneuploidy and genetic instability in cancer cells. Microtubule (MT)-generated forces drive the dynamic movements of centrosomes, chromosomes and MTs themselves and are essential players in the build-up of the mitotic bipolar spindle (see [1-3] for reviews). These forces are generated by the directional movement of motor proteins along MTs (reviewed in [4]) and depend on the dynamic properties of MTs [3]. The establishment of a proper spindle geometry and bipolar organisation requires a regulated balance between opposite directional forces exerted along growing MTs [5,6]. Given that improperly assembled or multipolar spindles can drive chromosome mis-segregation, there is a growing research focus on the mechanisms through which this balance is generated.

Phosphorylation-based signalling networks play key roles in orchestrating the concerted action of mitotic MT regulators. The mitotic kinase Aurora-A localises at centrosomes and along the mitotic spindle MTs; therein, it phosphorylates a variety of factors required for entry into mitosis, maturation and separation of centrosomes and mitotic spindle organisation (reviewed in [7,8]). Aurora-A is encoded by a cancer-associated gene that is amplified and/or overexpressed in several tumor types ([9,10], see [11,12] for recent reviews). Increased Aurora-A protein abundance can also be determined at the post-transcriptional level through various mechanisms, including the interaction with stabilising factors ([13-15]; see [16] for a review). Aurora-A overexpression can induce aneuploidy through various routes in different cellular contexts, including centrosome amplification ([17]; reviewed in [18]), faulty cell division [19] or weakened mitotic checkpoint activity [20]. Aurora-A is therefore intrinsically oncogenic and it is regarded as a potential target in anti-cancer therapy (reviewed in [11,21]). Novel molecules with Aurora-A-inhibitory activity are being designed in many laboratories, some of which have yielded promising results in pre-clinical studies and are under evaluation in phase I and II clinical trials (see [12] for a recent review). These observations highlight the importance of unraveling Aurora-A downstream processes. Furthermore, they call for increasing attention on elucidating potentially adverse consequences of Aurora-A inactivation on cell division.

We previously noticed that RNA interference (RNAi)-mediated inactivation of Aurora-A in human cells yields the formation of multipolar spindles, that originate from MT-dependent fragmentation of the pericentriolar material (PCM) and are not associated with centrosome amplification [22,23]. Multipolar spindles also form in cells injected with anti-Aurora-A antibodies [24] or treated with the Aurora-A specific inhibitor MLN8054 [25]. These lines of evidence indicate that Aurora-A is required for maintenance of spindle pole integrity. An analysis of spindles with fragmented poles in Aurora-A-silenced (thereafter indicated as Aurora-Ai) cells revealed that there was an altered localisation of the MT regulators ch-TOG (colonic and hepatic tumor over-expressed gene) and MCAK (mitotic centromere-associated kinesin) at spindle poles [23]. MCAK is a MT-depolymerising kinesin [26], whereas ch-TOG is a MT-stabilising factor with MT-polymerising activity (reviewed in [27]) that regulates the overall MT dynamics in human cells [28,29]. MCAK and ch-TOG antagonistic functions need be accurately balanced to ensure proper spindle pole organisation [30]. We previously reported that RNAi-mediated inactivation of Aurora-A yielded an abnormal accumulation of ch-TOG at spindle poles. Furthermore, the concomitant inactivation of ch-TOG restored spindle pole cohesion in Aurora-Ai cells [23]. These findings and the observation that PCM fragmentation is MT-dependent [23], suggest that effects of Aurora-A inactivation on the spindle pole structure implicate an altered MT dynamics. However, the functional connection between Aurora-A, the activity of MT-regulatory factors and the structural organisation of spindle poles remains to be yet clarified mechanistically.

Here we have investigated the dynamic properties of MTs in Aurora-A-defective mitoses and show that they are hyperstable, a feature that is reversed by concomitant inactivation of ch-TOG. We then devised several unrelated strategies to decrease pole-directed spindle MT forces in cells in which Aurora-A was inactivated by either RNAi-mediated silencing or by the specific inhibitor MLN8237. We report that these independent approaches converge in preventing spindle pole disruption in Aurora-A-defective mitoses. These results therefore highlight a novel role of Aurora-A in modulating spindle MT-associated forces directed towards centrosomes, which ultimately regulate spindle pole cohesion. This newly emerging role places Aurora-A upstream of a novel mechanism that regulates the balanced segregation of chromosomes along a bipolar spindle axis.

## Methods

### Cell cultures, synchronisation protocols and treatments

U2OS cells (ATCC: HTB-96) were cultured as described [23]. For synchronisation, cells were subjected to a 24 hours block in 2 mM thymidine. Cultures were then released from the G1/S arrest by washing away the thymidine and adding fresh medium containing 30 μM deoxycytidine; about 10 hours post-release (see Figures) mitoses-enriched cultures were fixed and processed for immunofluorescence (IF) as described below. MLN8237 (20 or 50 nM, as indicated; Selleck Chemicals, Houston, TX, USA) and monastrol (MON, 100 μM; Biomol International, PA, USA) were added to thymidine-released cultures at the indicated times before harvesting. When indicated, asynchronously growing cultures were treated with MON for 6 hours; cultures were then washed three times in warm medium and released in complete medium for 30 and 60 minutes. For MT depolymerisation experiments, cells were incubated on ice for 10, 15 and 20 minutes, then fixed and processed for IF.

### RNAi

cDNA sequences targeted by small interfering (si)RNA oligonucleotides (QIAGEN, Hilden, Germany or Applied Biosystems/Ambion, Austin, TX, USA) are: 725-ATGCCCTGTCTTACTGTCA-743 (Aurora-A), 126-GAGCCCAGAGTGGTCCAAA-144 (ch-TOG), 397-GCATGCCGTGAAACGTATA-415 (Nuf2, kindly provided by P. Meraldi; described in [31]). A GL2 siRNA duplex targeting the luciferase gene was used for control. Final concentrations of siRNA oligonucleotides were: 80 nM (Aurora-A and GL2), 40 nM (ch-TOG) and 60 nM (Nuf2). In cotransfections of Aurora-A and either ch-TOG or Nuf2 siRNAs the final amount of siRNA oligonucleotides in control cultures was balanced (to 120 or 140 nM, respectively) by adding GL2 oligo. Transfection reagent was Oligofectamine (Invitrogen, Carslbad, CA, USA). When experiments were carried out in synchronous cultures, siRNAs were transfected at the moment of thymidine treatment. Cultures were analysed 48 hours after transfection (asynchronous cultures) or at the indicated times after thymidine release (synchronised cultures). Experiments were repeated 2 to 4 times; statistical analysis of data was performed using the χ^2 ^test and calculating standard deviations (s.d.), as indicated.

### IF

Cells grown on coverslips were fixed as follows: (a) -20°C methanol, 6 minutes; or (b) 4% PFA plus TritonX-100 0.2% in Pipes pH 6.9 20 mM, MgCl_2 _1 mM, EGTA 10 mM, 10 minutes at room temperature. Blocking and all antibody incubations were performed at room temperature in PBS/0.05% Tween 20/3% BSA. Cells were counterstained with 4,6-diamidino-2-phenylindole (DAPI, 0.05 μg/ml) and mounted using Vectashield (Vector Laboratories, Burlingame, CA, USA). Primary antibodies were: anti-alpha-tubulin (1:2 000, B-5-1-2, Sigma, St Louis, MO, USA), anti-Aurora-A (0.5 μg/ml, BD Transduction Laboratories, Franklin Lakes, NJ, USA), anti-phospho-Aurora-A (Thr288) (C39D8; Cell Signaling Technology, Danvers, MA, USA), anti-pericentrin (2 μg/ml, ab4448; Abcam, Cambridge, UK), anti-ch-TOG (1:25, ab18320, Abcam), anti-Nuf2 (1:300, kind gift of V. Draviam, described in [31], or 5 μg/ml, ab17058, Abcam). Samples were analysed using a Nikon (Tokyo, Japan) Eclipse 90i microscope equipped with a Qicam Fast 1394 CCD camera (Qimaging, Surrey, BC, Canada). Image acquisition and deconvolution on 0.6-μm Z-serial optical sections were performed using NIS-Elements AR 3.2 (Nikon); images were processed with Adobe Photoshop CS 8.0 and NIS-Elements AR 3.2.

### Western immunoblotting (WB)

Cells were lysed in RIPA buffer (50 mM Tris-HCl pH 8.0, 150 mM NaCl, 1% NP40, 1 mM EGTA, 0.25% sodium deoxycholate) supplemented with protease and phosphatase inhibitors. Proteins were resolved by electrophoresis on 10% Laemmli gel and transferred on a nitrocellulose membrane (Protran BA83, Whatman, Kent, UK) using a semi-dry system (BIO-RAD, Hercules, CA, USA). 30 μg of extract per lane were loaded. Blocking and antibody incubations were performed at room temperature in PBS/0.1% Tween 20/5% low fat milk. Antibodies were: anti-Aurora-A (0.5 μg/ml), anti-Nuf2 (1:1 000, kind gift of V. Draviam), anti-actin (0.5 μg/ml, I-19; SantaCruz Biotechnology, Santa Cruz, CA, USA). Signals were visualized by enhanced chemiluminescence detection (ECL plus, GE Healthcare, Waukesha, WI, USA, and Protein Detection System, GeneSpin, Milan, Italy).

## Results

### Aurora-A inactivation yields hyperstabilisation of mitotic spindle MTs

**Figure 1:**
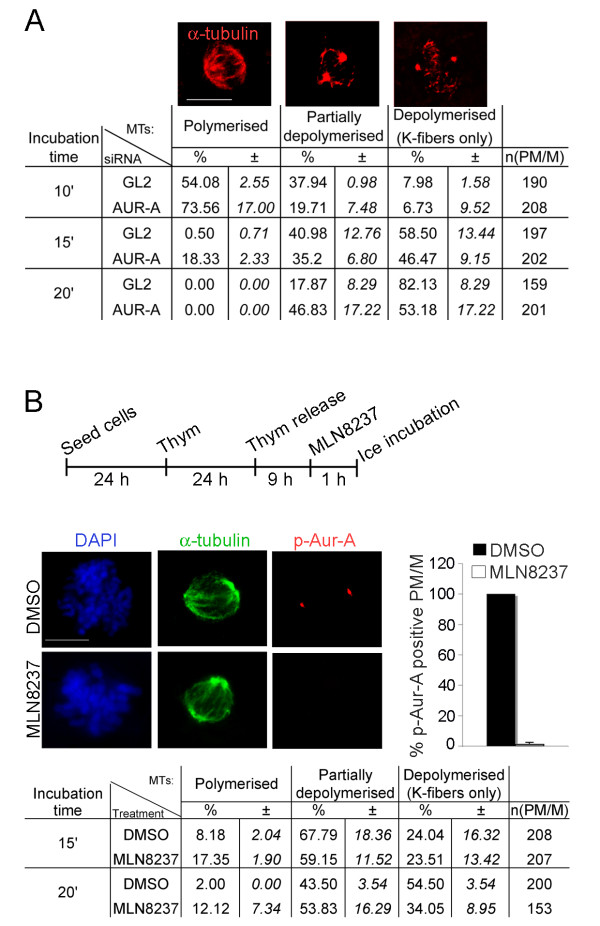
**Aurora-A inactivation induces hyperstabilisation of spindle MTs**. **A. **PM and M cells from control (GL2) and Aurora-Ai cultures incubated on ice for the indicated times were classified according to the status of MTs, examplified in the IF panels. The rightmost column indicates the number (n) of scored PM/M cells in 2 independent experiments; mean values (%) and s.d. (italics) are indicated. **B. **The protocol of MLN8237 treatment in synchronous cultures is shown (time intervals not represented to scale). Histograms show the percentage of PM and M cells displaying active pThr288-Aurora-A (p-Aur-A-positive) in control (DMSO) or MLN8237-treated cultures (200 counted cells per condition, 2 experiments; error bars represent s.d.); representative images are shown in the IF panels. In the table below, DMSO- or MLN8237-treated cells subjected to the ice-induced depolymerisation assay were classified as in A (2 independent experiments). Scale bars: 10 μm.

Aurora-A can be effectively silenced by RNAi in human cells, providing a useful tool to unravel the biological functions of this kinase [22-24,32]. In our assays, transient transfection of Aurora-A-specific siRNA oligonucleotides (48 hours) in human U2OS osteosarcoma cell cultures routinely reduced the overall Aurora-A protein level by about 90% of that measured in control cultures by WB assays. By single-cell IF analysis, Aurora-A signals were virtually undetectable or extremely reduced in over 80% of Aurora-Ai mitotic cells [22,23]. A significant fraction of these Aurora-Ai mitoses displayed spindles with multiple poles (indicated thereafter as "extrapoles") [22,23], which were abolished by co-inactivating the MT-stabilising factor ch-TOG in Aurora-Ai cells. We therefore wondered whether the dynamic properties of the spindle MTs are altered when Aurora-A function is disrupted. MT-stabilising and MT-depolymerising factors influence the rate of MT growth and shrinkage, which together determine the overall dynamic instability of MTs. Assaying the sensitivity of MTs to depolymerisation at low temperatures, in the presence or absence of a specific protein, can be used to assess the "protective" effect conferred by that factor, or absence thereof. We first investigated the sensitivity of MTs to ice-induced depolymerisation in a time-course analysis in Aurora-Ai cultures. Control cultures were interfered with neutral siRNAs, indicated as GL2i. After 48 hours of RNAi, cultures were incubated for 10, 15 or 20 minutes on ice and the status of MT polymerisation was examined in prometaphase (PM) and metaphase (M) cells (Figure 1A). In control GL2i cultures, mitotic cells underwent gradual MT disassembly during cold treatment: PM/M cells with polymerised MTs disappeared, paralleled by a gradual decrease over time of a category defined as "partially depolymerised" spindles, showing sparse, disorganised, yet still visible centrosome-originating MTs; we found a corresponding increase of the fully "depolymerised" category, in which centrosomes were totally devoid of MTs and alpha-tubulin was almost completely diffuse, with only stably kinetochore (KT)-attached MTs (K-fibers) remaining visible (exemplifying panels are shown in Figure 1A). After 20 minutes on ice the fully depolymerised actually became the almost exclusive or largely predominant phenotype in GL2i controls (over 80% of all PM/M). In Aurora-Ai PM/M, spindle MTs were significantly more resistant and their depolymerisation kinetics was slower compared to control cells (Figure 1A). Similar results were obtained by separately comparing cells in either the PM or the M stage, confirming a consistently slower cold-induced depolymerisation kinetics in Aurora-Ai compared to GL2i samples in each stage (not shown): thus, the increased resistance of MTs in Aurora-Ai cells is not due to an increased number of K-fibers but, rather, to an abnormal stability of non-KT MTs. Aurora-Ai mitoses are delayed at the PM stage [22] and in principle it was possible that the changes in MT dynamics were a mere side-effect of the abnormally long permanence of cells in this mitotic phase. We therefore devised an independent approach to analyse MT dynamics in Aurora-A-inactivated cells immediately after entering the first mitotic division. To this aim we pre-synchronised cultures at the G1/S transition, then allowed cells to progress synchronously to mitosis; 1 hour before harvesting the cells, we treated cultures with a recently developed specific Aurora-A inhibitor, MLN8237 [33], a second generation ATP-competitive molecule currently in phase I-II clinical trials [12]. Under these conditions Aurora-A is inactive, as shown by the disappearance of Thr-288-phosphorylated, i.e. active, Aurora-A (Figure 1B, IF panels and histograms). We then performed the ice-induced depolymerisation assay and fixed cells for IF analysis (see protocol in Figure 1B). We again observed a slower ice-induced MT depolymerisation in Aurora-A-inactivated compared with control cultures (Figure 1B): polymerised spindles were still present in 10-20% of Aurora-A-defective PMs after 15 and 20 minutes of ice-incubation while complete MT depolymerisation was achieved with significant slower kinetics compared with controls. This observation confirms that Aurora-A activity is required for normal MT dynamics at prometaphase.

### The induction of spindle extrapoles in Aurora-Ai mitoses is associated with ch-TOG-dependent MT hyperstabilisation

**Figure 2:**
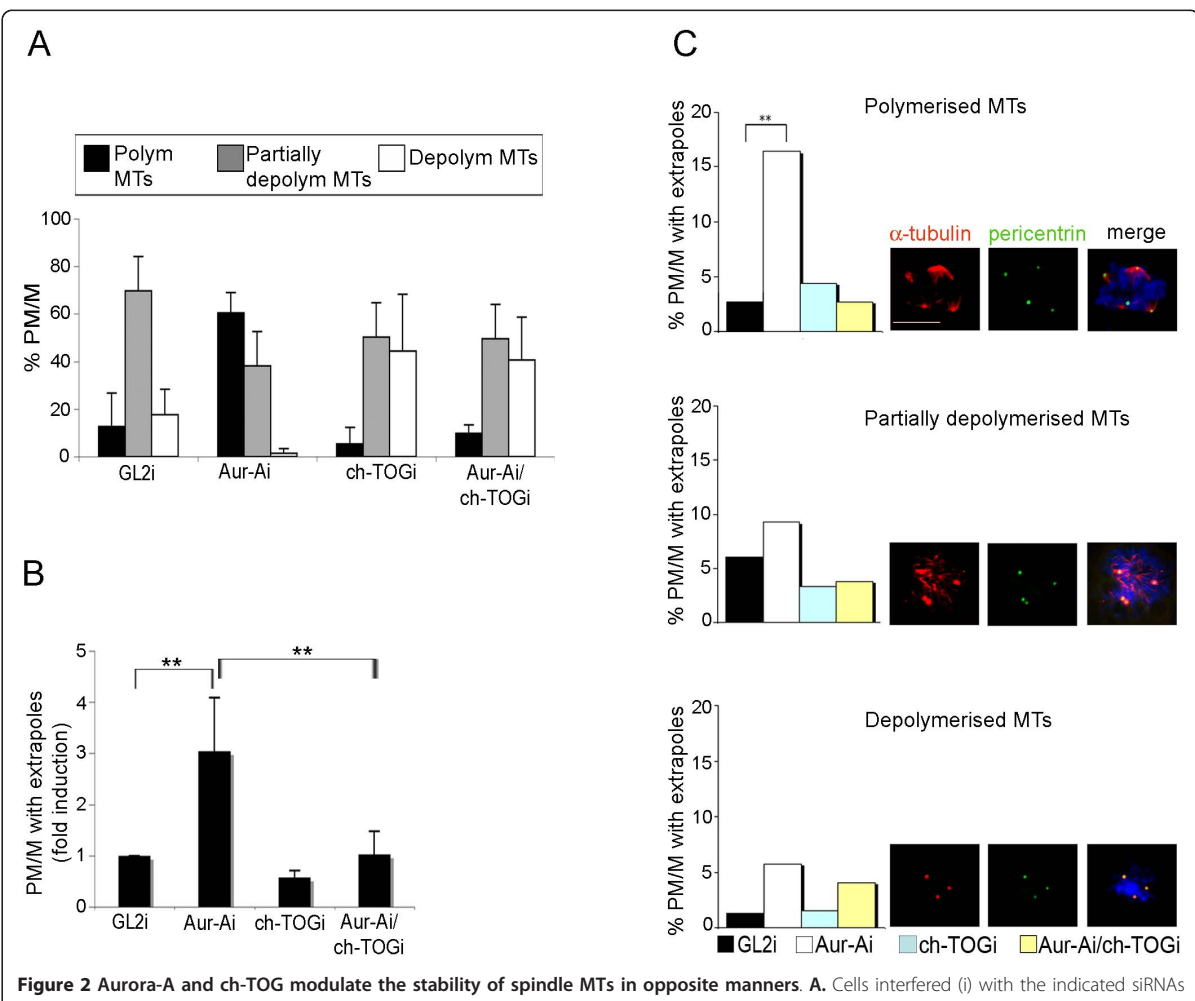
**Aurora-A and ch-TOG modulate the stability of spindle MTs in opposite manners**. **A. **Cells interfered (i) with the indicated siRNAs were incubated on ice for 15 minutes. Histograms represent the distribution of PM/M in the three MT categories identified in Figure 1A (at least 350 counted cells per condition, 2 experiments). **B. **PM/M with extrapoles were quantified (fold induction) in cultures interfered as indicated and incubated for 15 minutes of ice (at least 600 counted cells per condition, 2 experiments). **C. **Histograms represent the distribution of mitoses with fragmented spindle poles among the MT stability categories shown in Figure 1A (examples are shown in the IF panels). Around 700 PM/M were counted for each interference (2 experiments). Error bars represent s.d. **: p < 0.001, χ^2 ^test. Scale bar: 10 μm.

We next assessed whether co-inactivation of ch-TOG, which inhibits spindle pole fragmentation [23], also modifies the properties of MTs in Aurora-Ai cells. We used an RNAi protocol yielding efficient depletion of both Aurora-A and ch-TOG proteins in U2OS cells [23], then examined the status of MTs in double-interfered (Aurora-Ai/ch-TOGi) compared with controls (GL2i) and with single-interfered (either Aurora-Ai or ch-TOGi) PM and M cells in ice-induced depolymerisation assays (15 minutes of incubation) (Figure 2A). ch-TOGi PM/Ms showed effective ice-induced MT depolymerisation, with a somewhat higher frequency of the fully depolymerised category compared to GL2i controls, which is consistent with the loss of the MT-stabilising function of ch-TOG in these cultures. Furthermore ch-TOG co-inactivation in Aurora-A-defective cultures (ch-TOGi/Aurora-Ai) reduced the MT hyperstabilisation caused by Aurora-A inactivation alone and restored sensitivity to ice-induced depolymerisation (Figure 2A). In parallel, ch-TOG inactivation inhibited spindle pole fragmentation in Aurora-Ai ice-incubated mitoses (Figure 2B). Therefore, Aurora-A silencing induces the appearance of spindles with fragmented poles, abnormal ch-TOG accumulation at poles [23] and hyperstabilisation of spindle MTs (Figure 1). Concomitant with this, ch-TOG activity is itself required for both spindle pole fragmentation ([23] and Figure 2B) and for increased MT stability (Figure 2A) in Aurora-Ai mitoses.

If the disruption of mitotic spindle poles induced by Aurora-A inactivation is not just concomitant with, but is functionally linked to, the induction of hyperstable MTs, then the "extrapole" phenotype in Aurora-Ai cultures should be characteristically associated with spindles composed of ice-resistant MTs. To test whether this was the case, we examined how PM/M cells with fragmented poles were represented within the MT categories described in Figure 1A after ice incubation. Pole fragmentation was particularly represented (~16%, representing a highly significant 5-fold induction compared to controls) among Aurora-Ai PM/M with ice-resistant MTs (Figure 2C). By contrast, no statistically significant occurrence of pole fragmentation was detected among Aurora-Ai mitoses with partially or fully depolymerised MTs compared with controls. ch-TOGi/Aurora-Ai PM/M cells tended to have normal bipolar spindles (see Figure 2B), and those rare cells that displayed spindles with extrapoles showed no specific association with the MT status, being uniformly distributed among the different categories, similar to multipolar mitoses occurring in control cultures (Figure 2C). We conclude that the absence of Aurora-A yields spindle pole fragmentation in those mitoses in which MTs are hyperstabilised.

### Eg5 is required for spindle pole fragmentation induced by Aurora-A inactivation

The dynamic activity of MTs at poles is important to counteract centrosome-directed MT forces that pull spindle poles apart during spindle assembly in PM [1,4]. The evidence that spindle pole fragmentation in Aurora-Ai mitoses only occurs in the presence of MTs [23], and preferentially in PM/M cells harbouring hyperstable MTs (Figure 2), suggests that in the absence of Aurora-A spindle poles may be subjected to excessive MT-originating pressure that eventually causes them to fragment. If that hypothesis is correct, inactivation of centrosome-directed forces should inhibit spindle pole disruption in Aurora-A-defective cells.

**Figure 3:**
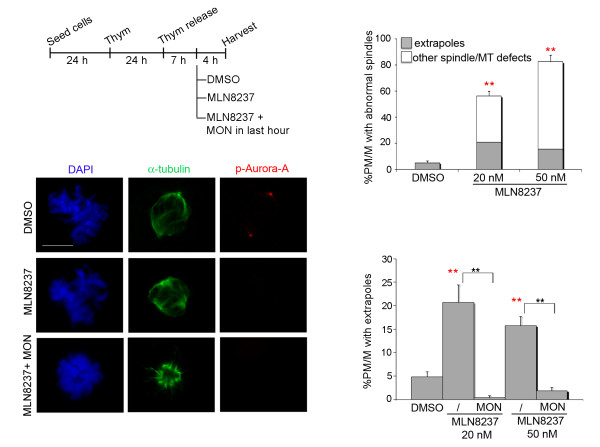
**Eg5 inhibition counteracts the induction of spindle pole fragmentation by Aurora-A inactivation**. The protocol to inhibit Aurora-A by MLN8237 in cells progressing towards mitosis is depicted (time intervals not represented to scale). Control cultures were treated with solvent (DMSO) in the same time window. When indicated, MON was added 1 hour before harvesting. Note the absence of active phosphorylated (pThr288) Aurora-A (in red in IF panels) in cells treated with MLN8237. Upper histograms represent the percentage of all spindle and MT abnormalities in control and MLN8237-treated cultures (200 counted PM/M per condition in 2 experiments); the grey fraction of the histograms represents mitoses with spindle extrapoles, while other defects (monopolar or disorganised spindles, few and short MTs) are in white. Lower histograms and IF panels show that concomitant Eg5 inhibition by MON prevents MLN8237-induced spindle pole fragmentation (note the failure of centrosome migration reflecting Eg5 inactivation in lower IF panels). 200 PM/M per condition were counted in 2 experiments. Error bars represent s.d. **: p < 0.001, χ^2 ^test. Red asterisks indicate significant differences with respect to DMSO controls, and black asterisks significant differences between Aurora-Ai mitoses with active or inactive Eg5. Scale bar: 10 μm.

A prominent contribution to centrosome-directed forces is provided by the plus-end-directed kinesin Eg5 along antiparallel MTs [4]. Eg5 activity is specifically inhibited by MON [5]. To assess whether Eg5 affects spindle pole fragmentation caused by Aurora-A inactivation, we used MON to inhibit Eg5 in combination with the MLN8237 Aurora-A inhibitor. We pre-synchronised U2OS cultures by thymidine arrest and release, then treated released cultures during progression from S- to M-phase with MLN8237, with or without MON, and analysed mitotic spindle organisation (Figure 3). In these experiments, MLN8237 treatment lasted 4 hours and yielded the induction of spindle abnormalities (Figure 3, upper histograms): these included multipolar spindles, spindles with disorganised MTs often focussed in multiple foci, some monopolar spindles and occasionally PMs with few and short MTs. Spindles with multiple poles represented 15-20% of all PM/Ms in MLN8237-treated cultures (Figure 3, IF panels and lower histograms), comparable to the occurrence in Aurora-Ai cultures. When MON was added, Eg5 was inhibited (as revealed by the presence of monopolar spindles in over 80% of control mitoses), and the generation of spindles with fragmented poles in MLN8237-treated cultures was abolished (Figure 3), with a parallel increase in monopolar figures (see example in the lower IF panels).

**Figure 4:**
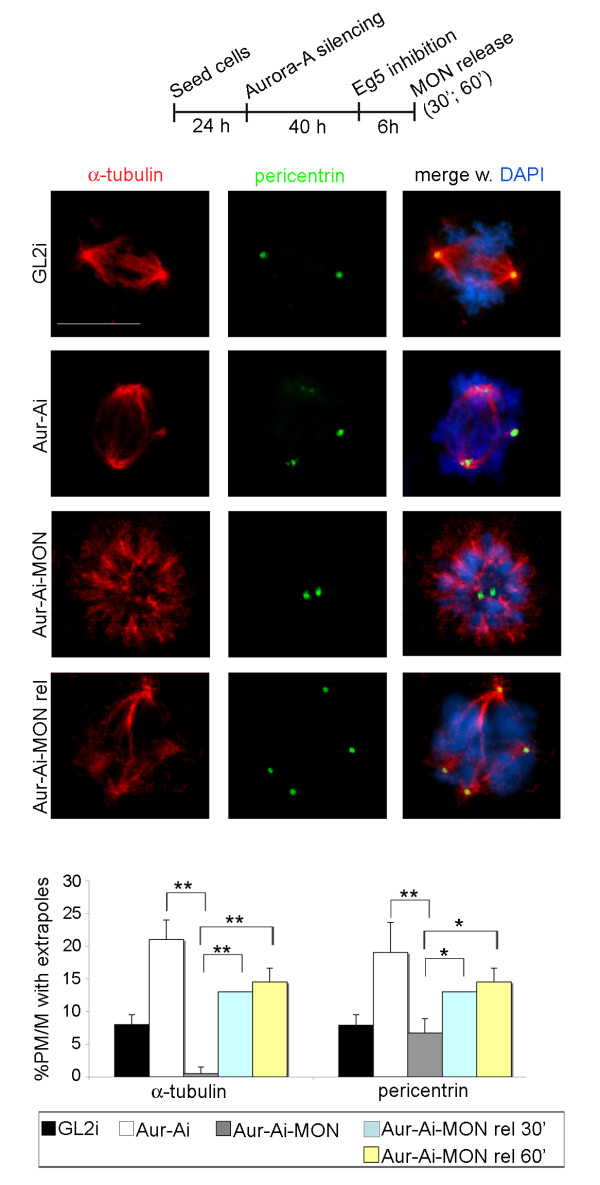
**Spindle pole fragmentation in Aurora-Ai mitoses depends on Eg5 activity**. A schematisation of the protocol is shown (time intervals not represented to scale). IF panels show spindles displaying normal or fragmented poles in control and Aurora-Ai cells, respectively (first and second row); monopolar spindles in Aurora-Ai cells treated with MON (third row); spindles displaying pole fragmentation in Aurora-Ai cells after MON release (MON-rel; lower row). Histograms represent the percentage of PM/M displaying fragmented poles, as assessed by alpha-tubulin (left) and pericentrin (right) staining (200 to 400 counted cells per condition in 2-4 experiments; s.d are shown). *: p < 0.01, **: p < 0.001, χ^2 ^test. Scale bar: 10 μm.

To further strengthen these results, we also performed MON treatment in cultures continuously subjected to Aurora-A RNAi. Simultaneous staining of MTs and pericentrin showed that MON treatment of Aurora-Ai cells inhibited PCM fragmentation and formation of spindle extrapoles compared with Aurora-Ai cells with active Eg5 (Figure 4). To further pinpoint the importance of Eg5-mediated forces in the generation of fragmented poles, we took advantage of the reversibility of MON to restore Eg5 activity in Aurora-Ai mitoses after MON wash-out (Figure 4). Eg5 activity was rapidly resumed on addition of MON-free medium, as monitored by the recovered ability of centrosomes to move apart. Concomitant with Eg5 reactivation, spindle pole fragmentation rapidly returned to comparable levels to those seen in Aurora-Ai cultures. Thus, restoring Eg5 activity was sufficient to generate the fragmentation phenotype induced by drug- or RNAi-mediated inactivation of Aurora-A.

The results thus far are consistent with the idea that directional spindle forces are imbalanced in Aurora-Ai mitoses, leading to pole fragmentation, and that proper balance is restored by reducing centrosome-directed MT forces. MT forces are therefore major players in the spindle pole fragmentation event caused by Aurora-A inactivation.

### Interfering with KT-MT attachments prevents spindle pole fragmentation induced by Aurora-A inactivation

In the experiments with MON, it was still possible that spindle formation in Eg5-inhibited PMs was blocked at a stage that preceded the Aurora-Ai-dependent fragmentation event. To distinguish between a specific temporal implication of Eg5 and a global alteration of spindle force orchestration in Aurora-A-defective cells, an alternative approach was needed to unbalance MT forces within the spindle.

**Figure 5:**
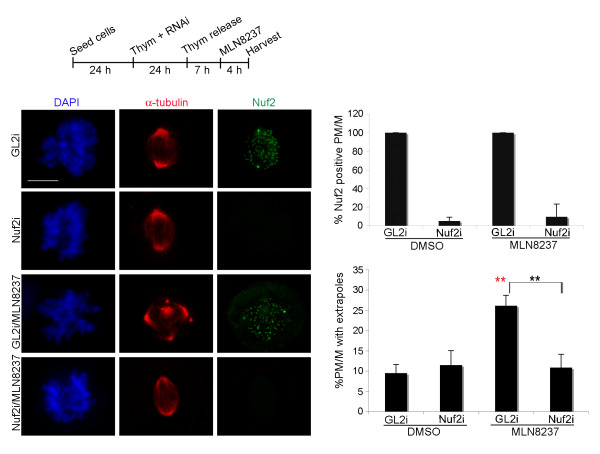
**Nuf2 silencing prevents MLN8237-induced spindle pole fragmentation**. A schematisation of the protocol for concomitant Nuf2 RNAi and Aurora-A inactivation (MLN8237) in cultures synchronously progressing from S-phase to mitosis is shown (time intervals not represented to scale). The efficiency of Nuf2 depletion following the RNAi protocol is quantified in the upper histograms (Nuf2 staining; 100-200 counted cells per condition in 2 experiments; see representative images in IF panels). Lower histograms (PM/M cells with fragmented poles by alpha-tubulin staining; 150-250 counted cells per condition, 2 experiments) and IF panels show that generation of extrapoles is reduced by Nuf2/Aurora-A co-inactivation compared to Aurora-A inactivation alone. **: p < 0.001, χ^2 ^test. Red asterisks indicate significant differences with respect to DMSO controls, and black asterisks significant differences between MLN8237-treated mitoses with (GL2i) or without (Nuf2i) the Nuf2 protein. s.d are shown. Scale bar: 10 μm.

**Figure 6:**
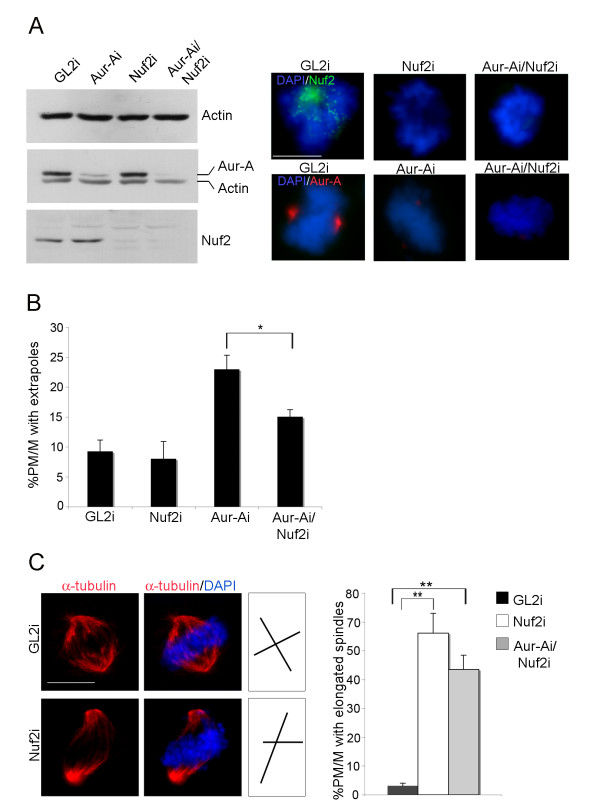
**Spindle pole fragmentation in Aurora-Ai mitoses depends on active Nuf2**. **A. **The efficiency of Aurora-A and Nuf2 depletion after RNAi was assessed by WB (left panels) and IF (right panels) analyses. **B**. Histograms represent the percentage of PM/M displaying fragmented spindle poles (alpha-tubulin staining) after transfection with the indicated siRNAs (at least 200 cells per condition, 2 experiments). **C. **Elongated spindles induced by Nuf2 RNAi are not rescued in Aur-Ai/Nuf2i co-inactivated cells (about 200 counted PM/M per condition in 2 experiments). Spindle axes are schematised on the right: the pole-to-pole axis is longer in Nuf2i cells compared to controls. Error bars denote s.d. *: p < 0.01, **: p < 0.001, χ^2 ^test. Scale bar: 10μm.

During normal spindle assembly, K-fibers-generated forces facilitate centrosome separation at PM [34] and need counteracting by motor-associated MT-focusing activities at poles [35]. We reasoned that reducing KT-generated polewards forces can provide an alternative approach to unbalance spindle forces while leaving motors undisturbed. To achieve this, we set up RNAi protocols to inactivate the KT protein Nuf2 and thus destabilise KT-MT attachments [36] in U2OS cells, in combination with Aurora-A inactivation by either MLN8237 treatment in synchronous (Figure 5, IF panels and upper histograms) or RNAi-mediated silencing in asynchronous cultures (Figure 6A). Using both experimental conditions, the occurrence of PM/M displaying spindle extrapoles was significantly reduced in Nuf2/Aurora-A co-inactivated cultures compared to those defective for Aurora-A alone (Figure 5, lower histograms; Figure 6B). This indicates that K-fibers are involved in the generation of the pole fragmentation phenotype. Nuf2i typically yields the appearance of elongated mitotic spindles [35,36]. These elongated figures were unaffected by the simultaneous inactivation of Aurora-A (Figure 6C): thus, Aurora-A/Nuf2 co-inactivation does not necessarily restore the overall spindle shape or organisation, but specifically prevents spindle pole fragmentation. These results support the conclusion that, in the absence of Aurora-A, KT-generated MT forces are imbalanced at the level of the poles, causing excessive pressure on poles and the ensuing fragmentation of the PCM.

## Discussion

**Figure 7:**
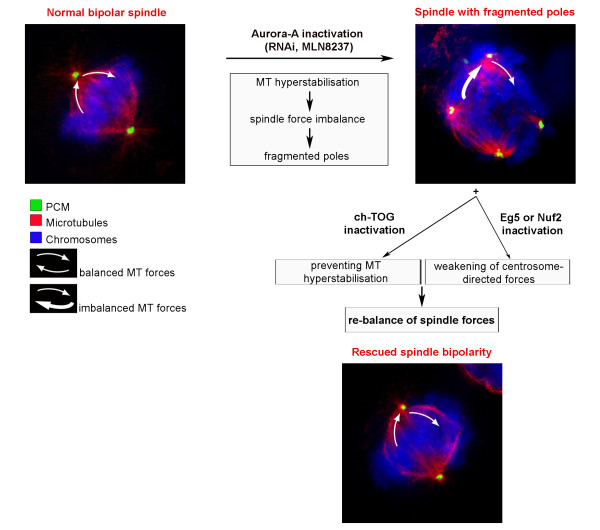
**Aurora-A modulates the balance of forces required for spindle pole integrity: a model**. Left upper panel: in a normal mitosis balanced MT forces determine the formation of a symmetrical bipolar spindle. Arrows represent opposite-directed MT forces. Right upper panel: in Aurora-A-defective mitoses, the spindle displays fragmented poles. Lower panel: spindle pole disruption is prevented by either inactivating a MT stabiliser (ch-TOG), or weakening KT-generated (Nuf2 silencing), or inhibiting motor-associated (Eg5 inhibition) centrosome-directed MT forces in the absence of Aurora-A activity. The model suggests therefore that spindle poles fragment consequently to the imbalance in MT-generated forces in Aurora-A-defective mitoses.

Among determinants of the spindle bipolar architecture, forces generated by motor proteins moving along antiparallel or astral MTs [1,4], as well as by K-fibers [34,35], have prominent roles. This work shows that Aurora-A acts upstream of these forces. Several lines of evidence demonstrate that in Aurora-A-defective cells (either RNAi-silenced or MLN8237-inhibited) an imbalance among MT forces within the spindle underlies the fragmentation of spindle poles. First, Aurora-A-defective mitoses display hyperstable MTs associated with spindle pole fragmentation, but co-inactivation of ch-TOG rescues the spindle pole fragmentation phenotype ([23] and this work) and concomitantly counteracts the abnormal MT hyperstabilisation caused by Aurora-Ai. Second, MON specifically inhibits the kinesin Eg5 and similarly attenuates the spindle pole fragmentation phenotype induced by either RNAi- or drug-mediated inactivation of Aurora-A. ch-TOG and Eg5 regulate independent functions during spindle assembly, respectively as a MT stabiliser and a MT plus-end-directed motor with crosslinking activity. The evidence that the loss of either activity prevents Aurora-A-dependent pole abnormalities, despite of their otherwise divergent biological functions, strengthens the idea that the rescuing activity is associated with their shared property to modulate pole-directed forces. Third, a similar inhibition was also observed after silencing of the KT protein Nuf2, under which condition MT-KT attachments are destabilised. The common feature of the "rescuing" conditions identified in this work is that they all decrease MT-mediated pressure on poles. These data integrate in the model illustrated in Figure 7, which schematises how Aurora-A interplays with forces regulating spindle pole formation: it is conceivable that in Aurora-A-defective cells the pressure directed towards poles is abnormally high, or is not properly counteracted at the PCM level, such that spindle poles fragment. Retrospectively, this model is consistent with our previous observation that the distance between the two main poles in Aurora-Ai PMs displaying extrapoles is higher than in bipolar spindles [23], a feature indicative of excessive outwards-directed spindle forces. Together these results indicate that Aurora-A is needed for establishing a finely-tuned balance among MT-associated forces operating in bipolar spindle formation.

Here we have selected ch-TOG, Eg5 and Nuf2 as paradigmatic examples of spindle regulatory activities and have manipulated their relative abundance or activity as experimental tools to perturb the equilibrium between MT forces. ch-TOG and Aurora-A are part of a complex in human cells [37] and in *Xenopus *extracts [38]. The *Xenopus *complex also contains Eg5 [38], and *Xenopus *Eg5 is a substrate of Aurora-A [39]. A likely possibility therefore is that Aurora-A regulates Eg5, and possibly ch-TOG, by modulating their interactions, and, in the case of Eg5, its phosphorylation status. Interestingly, ch-TOG has recently been identified in a proteome-wide search for potential Aurora-A substrates based on a computational integrated approach [40], although its actual phosphorylation by Aurora-A is not demonstrated. As an independent approach to restore the balance of forces within Aurora-Ai spindles, we have silenced Nuf2 and found that this also rescues the loss of spindle pole integrity in Aurora-A-defective mitoses. This finding does not necessarily imply a direct link between Aurora-A and the kinetochore protein Nuf2, but is more likely to reflect a global re-balancing of MT forces in Aurora-A-defective cells following the destabilisation of MT-KT attachments, adding support to the notion that K-fibers directly contribute to spindle pole organisation [34,35].

Finally, the present data raise a concern that Aurora-A inhibitors, currently under evaluation in anti-cancer therapy, may facilitate the formation of multipolar spindles and hence chromosome segregation defects. This issue deserves careful consideration because it may have opposite outcomes depending on the extent of chromosome imbalance and on the genetic background of cancer cells, e.g. for the status of tumor suppressors/apoptosis-inducing genes. On the one hand, mild aneuploidy facilitates tumorigenesis; on the other hand, massive aneuploidy generated by strong mis-segregation defects yields inviable cellular progeny and it is therefore regarded as a potential strategy to kill cancer cells ([41]; discussed in [42]). Investigating the long-term outcome of Aurora-A-inactivated mitoses with "extrapoles" will reveal whether they generate severely aneuploid inviable daughter cells, contributing to the therapeutic potential of Aurora-A inhibition, or on the contrary aneuploid viable cells, which would instead represent a detrimental consequence. Of relevance to this issue, we identify in this study two conditions that mitigate the multipolar phenotype induced by Aurora-A inhibition, i.e. inhibiting Eg5 activity or KT-MT attachments. Interestingly, inhibitors of either Eg5 or KT-MT interactions are also proposed as useful molecules in anti-cancer therapies [43,44]. An in-depth investigation of the interactions between these classes of molecules will be useful to devise effective therapeutic protocols.

## Conclusions

The present work uncovers a novel function of Aurora-A in orchestrating the balance between MT forces that ensure the proper organisation of spindle poles. This adds a novel aspect to the array of mitotic processes downstream of this multifaceted kinase. Aurora-A is frequently overexpressed in cancer and it is regarded as a promising target in anti-cancer therapies: our results draw attention on possible "off-target" effects of therapeutic approaches entailing the inactivation of the kinase. They also suggest that investigating the status of spindle forces in Aurora-A-overexpressing cells may be of relevance for our understanding of the mechanisms that underlie Aurora-A transforming ability.

## List of abbreviations

ch-TOG: colonic and hepatic tumor over-expressed gene; GL2i, Aurora-Ai, ch-TOGi, Nuf2i: GL2-, Aurora-A-, ch-TOG-, Nuf2- silenced by RNA interference; IF: immunofluorescence; KT: kinetochore; M: metaphase; MCAK: mitotic centromere-associated kinesin; MON: monastrol; MT: microtubule; PCM: pericentriolar material; PM: prometaphase; RNAi: RNA interference; siRNA: small interfering RNA; WB: western immunoblotting.

## Competing interests

The authors declare that they have no competing interests.

## Authors' contributions

IAA participated in the design of the project, performed the experiments, analysed the data and generated the figures. MG contributed to carry out the experiments and to perform statistical analysis of the data. PL participated in the design and coordination of the project and contributed to the writing of the manuscript. GG conceived and coordinated the project and drafted the manuscript. All authors have read and approved of the final version of the manuscript.
